# CB-GFL: carbon-budgeted federated learning with blockchain auditing for healthcare applications

**DOI:** 10.3389/fdgth.2026.1961099

**Published:** 2026-10-28

**Authors:** Tanisha Bhardwaj, K. Sumangali

**Affiliations:** School of Computer Science Engineering and Information Systems, Vellore Institute of Technology, Vellore, Tamil Nadu, India

**Keywords:** blockchain, carbon budget, cardiovascular disease, federated learning, green AI, healthcare AI, lightgbm, Matthews correlation coefficient

## Abstract

Artificial intelligence (AI) in healthcare has made it harder to protect patient data, prediction performance, and environmental sustainability in federated learning (FL) systems. A single platform for sustainable healthcare applications, Carbon-Budgeted Green Federated Learning (CB-GFL), merges carbon-aware client selection, blockchain-based auditing, and energy-efficient collaborative learning. The framework integrates (i) a carbon-budget-aware client selection strategy formulated as a 0–1 knapsack optimisation problem with a greedy approximation guarantee of approximately 63.2%; (ii) a blockchain auditing layer consisting of three smart contracts, the Carbon Budget Ledger, Energy Verification Contract, and Incentive/Penalty Contract, secured using SHA-256 hash-based immutable records; and (iii) an ensemble learning model combining LightGBM, XGBoost, and Gradient Boosting with SMOTEENN resampling. All experiments are conducted in a simulated federated environment, with clients executed on a single machine. CB-GFL achieves AUC = 0.9113 on the UCI 4-site benchmark (4 hospitals, 3 countries, *n* = 920), exceeding the 0.90 threshold, and AUC = 0.7138 on Framingham, consistent with the clinical gold standard of 0.70 to 0.75. In the balanced operating point, experimental findings show 83.61% classification accuracy, 82.34% weighted F1-score, 0.7138 AUC-ROC, and 0.2771 MCC, and 72.07% sensitivity in the screening configuration. The proposed system ensures 100% carbon-budget compliance and a 74-block tamper-resistant blockchain audit trail for energy responsibility. Comparison to 11 benchmark algorithms shows that the dataset's realistic predictive qualities are 0.14–0.29. Additionally, five-fold cross-validation shows steady generalisation with an average accuracy of 84.25% ± 1.03%. The results show that CB-GFL supports sustainable AI adoption and privacy-preserving, carbon-aware, auditable federated healthcare intelligence.

## 1 Introduction

Healthcare has advanced with artificial intelligence, detecting arrhythmias (1), interpreting chest x-rays (2), and predicting cardiac contractile failure before clinical signs (3). Despite these advances, three interdependent challenges inhibit large-scale AI deployment in clinical settings: patient privacy, high predictive performance, and environmentally sustainable model training. There are very few solutions that combine privacy, accountability and carbon efficiency in distributed healthcare systems.

A. Patient Data Privacy

Strict frameworks, such as HIPAA in the USA and GDPR in the EU, are in place that limit the sharing of patient information across institutional borders (4). In general, healthcare organisations keep data in isolated data repositories, and large and varied datasets for training of machine learning models are lacking. The limited demographic, disease and generalizability of the data used to train models at one institution can lead to difficulties in generalizability. Federated Learning (FL) is a promise on collaborative model training without patient data translation (6). Recent surveys confirm FL as the leading privacy-preserving paradigm for smart healthcare (25), with growing adoption across cardiovascular, oncology, and radiology applications (27). Local models are trained locally at FL institutions and only model parameters (or gradients) are shared with a central aggregation server. This strategy greatly improves privacy, although FL frameworks are still subject to inference attacks like gradient inversion and model reconstruction, which may disclose sensitive training information. Traditional FL systems do not normally track or control the impact on the environment of regular, distributed model training.

### 1.1 Environmental sustainability

Model creation and deployment demand more computing resources due to AI's fast growth. Large scale training of deep learning models can produce substantial carbon emissions, though this can be different depending on the local electricity generation mix (5, 14). In the case of healthcare applications, this is made even harder as the predictive models need to be continuously updated as clinical practice, demographic characteristics, and disease patterns evolve. Carbon emissions are not a “cost of computation” anymore. While some systems like Green AI have found solutions to minimize computational energy consumption (11), carbon-budget compliance typically is not enforced, monitored, or verified in federated learning systems during collaborative training.

### 1.2 Distributed trust and verification

In collaborative federated learning, multiple independent healthcare institutions can share computing resources, yet operate independently. In such places, energy and computational activity are self-reported, and it is not known if they are accurate. When the energy use and the carbon emissions cannot be substantiated, sustainability pledges and incentive programmes are ineffective and unreliable. Records of federated learning activities are kept using the blockchain technology, which is immutable, decentralised and tamper-proof. Blockchain and federated learning provide audits of carbon budget, carbon usage and incentive payments for participants without a trusted central authority.

### 1.3 Summary of contributions

- A Carbon-Aware Client Selection (CACS) strategy is proposed as a 0–1 knapsack optimization problem with an approximate greedy solution of 63.2% which allows for energy-efficient client selection under defined carbon-budget constraints, validated under binding budget experiments where high-emission clients are actively excluded and blockchain penalty contracts are triggered.
- The Carbon Budget Ledger (CBL), Energy Verification Contract (EVC), and Incentive/Penalty Contract (IPC) are developed as a blockchain-based auditing framework, which ensures an immutable SHA-256-linked record of carbon emissions, energy consumption, and incentive transactions throughout the federated learning process.
- A hybrid ensemble learning model combining LightGBM, XGBoost, and Gradient Boosting with SMOTEENN resampling is developed using an 84-feature clinical dataset from the Framingham Heart Study, achieving 83.61% accuracy, 82.34% weighted F1-score, 0.2771 MCC, and 0.7138 AUC-ROC under the balanced operating configuration.
- A comprehensive comparative evaluation involving eleven machine learning algorithms is conducted to analyze the achievable performance range on the Framingham dataset and to position the proposed framework within established clinical benchmark performance.
- Multiple clinical operating points are analyzed to support different deployment objectives, including high-sensitivity screening, balanced classification, and high-specificity clinical decision support.
- The models are also statistically validated comprehensively by running five-fold cross validation, bootstrapping the confidence intervals and Wilcoxon signed rank significance test with Bonferroni correction to test the robustness and generalization of the models.

### 1.4 Paper organization

The rest of the paper is organised as follows: Section 2 reviews work related to this. Section 3 develops the mathematical model, including all the equations and Algorithm 1. The three-layer architecture is described in section 4. The experimental setup is described in section 5. Section 6 presents results. Section 7 discusses the findings and their implications, and Section 8 concludes the research.

## 2 Literature survey

### 2.1 Federated learning for healthcare

The Federated Averaging (FedAvg) approach of McMahan et al. (6) allows to learn distributedly without sharing raw training data, while keeping communication costs low. Based on this approach, Rieke et al. (7) analysed federated learning systems from 71 healthcare institutions and found five significant challenges: data heterogeneity, communication efficiency, security, fairness, and regulatory compliance. They did not consider energy usage efficiency and an environmental footprint. In collaboration with others, federated learning could also segment brain cancer MRI images in a similar manner to centralised learning, as Sheller et al. (8) demonstrated, and could be an effective solution for collaborative medical imaging without compromising patient privacy. To combat the statistical heterogeneity present in distributed data, Li et al. (9) introduced FedProx, a method that includes a proximal regularisation term into the local optimisation objective, resulting in reduced client drift under non-IID data. Kaissis et al. (10) extended federated learning to medical imaging with differential privacy, which ensured privacy at the expense of predictive performance. Recently, Dayan et al. (23) developed a federated learning system for the COVID-19 prognosis from data collected from seven US healthcare institutions with an AUC of 0.93. These studies demonstrate privacy-preserving and collaborative model development capabilities of federated learning, but none of them explore carbon-aware training, energy accounting, or sustainable mechanisms for verifiable sustainability in the context of healthcare AI.

### 2.2 Green AI and sustainable computing

Barbierato and Gatti (11) introduced Green AI, which adds computational efficiency and environmental sustainability as two criteria to evaluate AI, besides predictive performance. Lannelongue et al. (26) proposed GREENER principles for environmentally sustainable computational science, providing a framework for responsible AI development in healthcare research settings. Their research sparked a novel line of thought for energy use and carbon emissions in designing AI systems, not just on model performance. Additionally, Patterson et al. (14) showed that the carbon footprint of training machine learning models could differ significantly depending on the geographic location of machine learning data centres and the mix of electricity generation. This observation inspires the addition of carbon intensity parameters based on institutions in the proposed CB-GFL framework. Lannelongue et al. (12) came up with the Green Algorithms methodology to estimate the carbon emissions of computational workloads using hardware power consumption, execution time, and regional carbon intensity. This methodology is followed by the carbon estimation model used in this study which is presented in Equation (4). Moreover, Gao et al. (13) have shown that carbon-aware workload scheduling could cut down greenhouse gas emissions by around 30%–70% by optimizing the use of computational resources. Motivated by this idea, the proposed carbon-aware scheduling method for federated learning combines carbon-budget constraints into the client selection process, allowing for environmentally friendly collaborative model training.

### 2.3 Blockchain in distributed AI

In the context of the Industrial Internet of Things (IIoT), Zhou et al. (19) applied federated learning to blockchain to develop an incentive scheme for enhancing data quality in IIoT systems. While their system increased the accountability of participants and reliability of data, it failed to take into account monitoring of energy consumption or carbon emissions. In order to protect model integrity from poisoning attacks, Weng et al. (20) introduced DeepChain, a federated learning framework based on blockchain where model update hashes are stored on the blockchain. To ensure gradient exchange and anonymity during collaborative model training, Passerat-Palmbach et al. (21) proposed using blockchain smart contracts. According to this research, blockchain makes federated learning systems more data-aware, secure, and trustworthy. Blockchain has also been explored in clinical trial transparency (16) and healthcare record management (17). Azaria et al. (18) introduced MedRec, an early blockchain system for medical data access and permission management. Gonzales et al. (15) further demonstrated blockchain applications for outcomes research in sensitive healthcare domains including opioid treatment. TheR potential impact of distributed training on the environment has received little research attention, whereas blockchain-based federated learning models focus on privacy, secure model aggregation, and resistance to adversarial attacks. In contrast, the Carbon-Budgeted Green Federated Learning (CB-GFL) framework introduces a carbon accounting system, energy verification, and incentive management, providing a foundation for addressing sustainability in federated AI systems in the healthcare sector.

### 2.4 SOTA context and dataset comparison

The results on cardiovascular disease prediction should be interpreted after taking into account the different characteristics of datasets across studies published. A relatively well-balanced class distribution (1:0.84) and 303 samples were used to test several cardiovascular classification studies with AUC values of 0.92–0.94, and 13 clinical features. The Framingham Heart Study is more complex to predict because it has a larger sample size, more features, imbalance in classes, and a heterogeneous population. Performance indicators from the UCI Cleveland dataset are not similar to those from Framingham. Table 1 summarises the Framingham dataset's state-of-the-art (SOTA) performance as a benchmark.

**Table 1:** Framingham-specific SOTA comparison.

Method	AUC/C-statistic	Learning Setting	Remarks
Framingham Risk Score	0.70–0.75	Clinical risk prediction model	Widely used clinical benchmark for cardiovascular risk assessment
Logistic Regression	0.72–0.74	Centralized machine learning	Conventional baseline trained on pooled data
Random Forest	0.74–0.76	Centralized machine learning	Ensemble learning approach using centralized training
XGBoost with feature engineering	0.75–0.77	Centralized machine learning	Gradient boosting model with engineered clinical features
CB-GFL (Proposed)	0.7138	Federated learning across five simulated healthcare institutions	Privacy-preserving collaborative learning with blockchain-based carbon auditing

As shown in Table 1, CB-GFL's AUC of 0.7138 falls within the established Framingham performance range (0.70–0.77), achieved under privacy-preserving federated conditions across five simulated institutions. Because UCI Cleveland differs substantially in sample size, class balance, and feature composition, AUC values above 0.92 reported on that dataset are not directly comparable and are excluded from this benchmark comparison.

### 2.5 Research gap

Federated learning protects privacy, blockchain ensures trust, and Green AI reduces computational energy consumption. However, these capabilities are not yet fully integrated into a healthcare framework. The literature has neglected privacy-preserving federated learning, per-round carbon budget enforcement, blockchain-based energy usage verification, and green reward systems. Table 2 summarises research and showcases the CB-GFL framework's advantages over representational alternatives.

**Table 2:** Literature comparison of FL approaches.

Ref.	Method	Dataset/Application	Performance	Privacy-Preserving FL	Carbon Monitoring	Blockchain Auditing	Healthcare Application
(6)	FedAvg	MNIST, CIFAR	97.5%	✓	✗	✗	✗
(8)	Federated Medical Imaging	BraTS MRI	∼82%	✓	✗	✗	✓
(10)	Secure FL with Differential Privacy	Chestx-ray14	∼78%	✓ (Formal DP)	✗	✗	✓
(19)	Blockchain-enabled FL	Industrial IoT	∼76%	✓	✗	Partial	✗
(20)	DeepChain	MNIST	∼95%	✓	✗	✓	✗
(27)	Federated COVID-19 Prediction	Multi-hospital	∼88%	✓	✗	✗	✓
(11)	Green AI	Various AI Applications	N/A	N/A	Conceptual	✗	✗
Proposed	**CB-GFL**	**Framingham Heart Study**	**83.61%**	**✓**	**✓**	**✓**	**✓**

Bold values indicate the best-performing result in each column.

## 3 Mathematical framework

This section formulates the Carbon-Budgeted Green Federated Learning (CB-GFL) framework mathematically. To theoretically support the suggested system, the objective functions for carbon-aware client selection, federated optimisation, carbon emission estimate, blockchain-based energy auditing, and incentive allocation are developed. Algorithm 1 also covers the Carbon-Aware Client Selection (CACS) technique, which selects the best customers under carbon-budget limitations.

### 3.1 Global learning objective

Consider a federated healthcare network comprising *K* participating healthcare institutions. Each institution *k* maintains a private local dataset $D_{k}$ containing $n_{k}$ training samples. The objective of federated learning is to minimize the weighted global empirical loss while ensuring that patient data remain locally stored. The global optimization problem is formulated as

$$F(w)=\overset{K}{\underset{k=1}{\sum }}\frac{n_{k}}{n}F_{k}(w),$$ (1)

Where $n=\overset{K}{\underset{k=1}{\sum }}\;n_{k}$ and

$$F_{k}(w)=\frac{1}{n_{k}}\underset{(x_{i},y_{i})\in D_{k}}{\sum }\;\ell (\;f(w;x_{i}),y_{i}).$$

Here in equation (1), where $F_{k}(w)$ denotes the local empirical loss of institution *k*, ℓ(⋅) represents the classification loss function, $f(w;x_{i})$ denotes the model prediction for input sample $x_{i}$, and w represents the global model parameters. The global model is obtained by aggregating locally trained models according to their respective dataset sizes, thereby preserving data privacy while enabling collaborative model optimization.

### 3.2 Carbon-aware client selection

To promote environmentally sustainable federated learning, the proposed Carbon-Aware Client Selection (CACS) strategy selects participating institutions while satisfying a predefined carbon emission budget. Let $c_{k}$ denote the estimated carbon emission associated with institution *k*, and let θ represent the maximum allowable carbon budget for a communication round. The client selection problem is formulated as

$$\begin{array}{cccc} & \begin{array}{cc} \underset{S}{\max} & \underset{k\in S}{\sum }\;n_{k}\\ \mathrm{subject}\;\mathrm{to} & \underset{k\in S}{\sum }\;c_{k}\leq \theta , \end{array} & & \end{array}$$ (2)

As inferred from equation (2), where *S* denotes the subset of selected participating institutions. The optimization objective maximizes the total number of training samples contributed during each communication round while ensuring that the aggregate carbon emissions remain within the specified carbon budget. Since the 0-1 knapsack problem is NP-hard, a greedy approximation algorithm is employed that sorts clients in descending order of value-to-weight ratio, where value is the normalised sample count and weight is the estimated CO₂ emission per round. The greedy solution achieves an approximation ratio of ALG/OPT ≥ 1−1/e ≈ 0.6321, guaranteeing at least 63.2% of the optimal solution in the worst case. This bound follows from the fractional relaxation analysis of greedy knapsack algorithms (28). CB-GFL uses a computationally efficient greedy approximation method for the NP-hard classical 0–1 knapsack optimisation problem. After ranking institutions by carbon-efficiency ratio, customers are picked till the carbon budget is depleted. This approximation guarantees 63.2% of the ideal answer with minimal computing cost for large-scale federated healthcare implementations.

Algorithm 1 **Carbon-Aware Client Selection (CACS).**

**Table:** 

**Input:** Participating institutions $\Phi =\{\phi_{1},\phi_{2},\ldots ,\phi_{K}\}$, estimated carbon emissions $\left\{c_{k}\right\}$, local dataset sizes $\left\{n_{k}\right\}$, global carbon budget θ, and institution-specific carbon limits $\left\{B_{k}\right\}$.
**Output:** Selected client subset *S*.
1. Compute the carbon-efficiency ratio $r_{k}=\frac{c_{k}}{n_{k}}$ for each participating institution $\phi_{k}$.
2. Sort all institutions in ascending order according to $r_{k}$.
3. Initialize the selected client set as S=∅ and the accumulated carbon emission as $C_{\mathrm{total}}=0$.
4. For each institution $\phi_{k}$ in the sorted list, perform the following steps:
o If $C_{\mathrm{total}}+c_{k}\leq \theta$, $c_{k}\leq B_{k}$, and the remaining participation budget is available, then:
▪ Add $\phi_{k}$ to the selected set *S*.
▪ Update the accumulated carbon emission as
$C_{\mathrm{total}}=C_{\mathrm{total}}+c_{k}.$
5. If no institution satisfies the selection criteria (S=∅), select the three institutions with the largest local datasets as the fallback client set.
6. Update the estimated carbon emission for each selected institution using the exponential moving average (EMA):
${\hat{c}}_{k}=0.7\;{\hat{c}}_{k}+0.3\;C_{k}^{\left(t\right)}.$
Return the selected client subset *S*.

### 3.3 Fedavg via probability averaging

Because LightGBM, XGBoost, and Gradient Boosting models have different structures, direct parameter aggregation is not possible in the proposed CB-GFL framework. Aggregating the selected clients' class probability distributions yields the global model. The probability aggregation at communication round t+1 is defined as

$$\begin{array}{cccc} & P^{\left(t\;+\;1\right)}(x)=\underset{k\in S^{\left(t\right)}}{\sum }\frac{n_{k}}{n_{S^{\left(t\right)}}}\;P_{k}^{\left(t\right)}(x), & & \end{array}$$ (3)

where

$$n_{S^{\left(t\right)}}=\underset{k\in S^{\left(t\right)}}{\sum }\;n_{k}.$$

Here in equation (3), where $P_{k}^{\left(t\right)}(x)$ denotes the class probability vector predicted by client *k* during communication round *t*, $S^{\left(t\right)}$ represents the subset of clients selected for that round, and $n_{k}$ is the number of training samples maintained by client *k*. Client contributions are weighted by local dataset size to ensure that institutions with larger datasets have a proportional impact on the aggregated prediction. The final prediction is obtained by selecting the class with the highest aggregated probability, $\hat{y}=\arg\underset{c}{\max}\;P_{c}^{\left(t\;+\;1\right)}(x),$ followed by application of the predefined decision threshold corresponding to the selected clinical operating point. This probability-based aggregation strategy enables collaborative learning among heterogeneous machine learning models without requiring a common model architecture, thereby improving flexibility while preserving compatibility with federated learning.

### 3.4 Carbon emission model

To quantify the environmental impact of federated learning, the proposed CB-GFL framework estimates the carbon emissions generated by each participating institution during every communication round. Following the Green Algorithms methodology (12), the carbon emission associated with client *k* during communication round *t* is computed as

$$\begin{array}{cccc} & C_{k}^{\left(t\right)}=\left(\frac{P_{k}\;\tau_{k}}{3,600,000}\right)\times CI_{k}, & & \end{array}$$ (4)

As computed in equation (4), where $C_{k}^{\left(t\right)}$ denotes the carbon emission (kg $CO_{2}$ eq) generated by client *k* during round *t*, $P_{k}$ is the average power consumption of the training device (W), $\tau_{k}$ represents the local training time (s), and $CI_{k}$ is the regional electricity carbon intensity (kg $CO_{2}$ eq/kWh). The conversion factor 3,600,000 converts electrical energy from watt-seconds to kilowatt-hours. In this study, an electricity grid emission factor of 0.708 kgCO₂/kWh (Central Electricity Authority, Government of India, 2023) is used for all experiments. The carbon emissions of all participating clients are accumulated during each communication round and constrained by the predefined carbon budget θ, such that $\underset{k\in S^{\left(t\right)}}{\sum }\;C_{k}^{\left(t\right)}\leq \theta .\;$

The proposed Carbon-Aware Client Selection (CACS) strategy ensures that only clients that meet the carbon budget are engaged in the federated training process. To capture temporal variations in computational workload and energy consumption, the estimated carbon emission for each client is updated after every communication round using an exponential moving average (EMA):

$${\hat{c}}_{k}^{\left(t\;+\;1\right)}=\alpha {\hat{c}}_{k}^{\left(t\right)}+(1-\alpha )C_{k}^{\left(t\right)}\;$$ (5)

As shown in equation (5), where ${\hat{c}}_{k}^{\left(t\right)}$ is the estimated carbon emission for client *k* at communication round *t*, $C_{k}^{\left(t\right)}$ is the measured carbon emission during the current round, and α=0.7 is the smoothing factor that balances historical observations with recent measurements. Reliable carbon forecast and informed client selection in following federated learning rounds are improved by adaptive estimate.

### 3.5 Token incentive economy

A blockchain-enabled token incentive mechanism rewards institutions for carbon-budget compliance in the proposed CB-GFL framework to encourage environmentally responsible federated learning. Let $\rho_{k}^{\left(t\right)}=\frac{C_{k}^{\left(t\right)}}{B_{k}},$ denote the carbon utilization ratio of client *k*, where $C_{k}^{\left(t\right)}$ is the measured carbon emission during communication round *t* and $B_{k}$ represents the carbon budget allocated to that client. Based on the carbon utilization ratio, the token reward assigned to client *k* is defined as

$$T_{k}^{\left(t\right)}=\{\begin{array}{cc} +10, & \rho_{k}^{\left(t\right)}<0.5,\\ +5, & 0.5\leq \rho_{k}^{\left(t\right)}<0.8,\\ 0, & 0.8\leq \rho_{k}^{\left(t\right)}\leq 1.0,\\ -10, & \rho_{k}^{\left(t\right)}>1.0. \end{array}$$ (6)

Accordingly in equation (6), the institutions consuming less than 50% of their allocated carbon budget are classified as Green Champions and receive the maximum reward of 10 tokens. Institutions operating within 50%–80% of the allocated budget receive 5 tokens, while those utilizing 80%–100% of the budget remain compliant without receiving additional incentives. Institutions exceeding their allocated carbon budget incur a penalty of 10 tokens, thereby discouraging excessive energy consumption. The cumulative token balance of each participating institution throughout the federated learning process is computed as $\Lambda_{k}=\overset{R}{\underset{t=1}{\sum }}\;T_{k}^{\left(t\right)}$, where *R* denotes the total number of communication rounds.

### 3.6 Focal loss with class weighting

The Framingham Heart Study dataset has a class imbalance of 1:5.57 positive to negative coronary heart disease (CHD) patients. The proposed CB-GFL framework uses weighted focal loss with inverse-frequency class weighting and focal modulation to reduce majority class bias. Definition of loss function:

$$L_{FL}=-\overset{C}{\underset{c=1}{\sum }}\;w_{c}(1{\hat{y}}_{c}{)}^{\gamma }y_{c}\log({\hat{y}}_{c})$$ (7)

As shown in equation (7), where $y_{c}$ is the ground-truth label for class *c*, ${\hat{y}}_{c}$ denotes the predicted probability, *C* is the total number of classes, $w_{c}$ represents the class-specific weighting factor, and γ is the focal parameter. The inverse-frequency class weights are computed as $w_{c}=\frac{n}{C\;n_{c}}$ where *n* is the total number of training samples and $n_{c}$ denotes the number of samples belonging to class *c*. For the minority CHD class, the resulting weight is approximately 3.3, thereby assigning greater importance to underrepresented positive cases during model optimization. The focal parameter is set to γ=2, which reduces the contribution of well-classified samples while increasing the learning emphasis on difficult and misclassified minority-class instances.

## 4 System architecture

The Carbon-Budgeted Green Federated Learning (CB-GFL) system uses a three-layer hierarchical architecture to accomplish privacy-preserving collaborative learning, verifiable carbon responsibility, and sustainable resource use in dispersed healthcare contexts.

Figure 1 shows the Federated Learning Layer, Blockchain Audit Layer, and Edge Client Layer working together during each federated learning communication round. The Federated Learning Layer coordinates worldwide training. It selects carbon-aware clients using the proposed Carbon-Aware Client Selection (CACS) algorithm, distributes the current global model to selected healthcare institutions, aggregates probability predictions from participating clients, and updates the global model after each communication round. Before model aggregation, this layer continually checks that cumulative carbon emissions stay under the carbon budget. All carbon-related and training events are recorded decentralised and tamper-resistant via the Blockchain Audit Layer. The Carbon Budget Ledger (CBL), Energy Verification Contract (EVC), and Incentive/Penalty Contract (IPC) smart contracts monitor carbon emissions, verify energy use, and administer token-based incentives for collaborating organisations. Throughout the federated learning lifecycle, SHA-256 hash creation secures and links each transaction to the preceding block, assuring data integrity, traceability, and transparent auditing. Dispersed healthcare institutions at the Edge Client Layer keep patient data locally and train models without sharing clinical data. Each institution preprocesses its local dataset, trains the heterogeneous ensemble model, calculates carbon emissions, and transmits the federated server encrypted probability predictions and certified energy use records. Collective learning is only allowed if institutions' estimated carbon emissions satisfy CACS algorithm carbon-budget restrictions. These three layers enable the CB-GFL system to incorporate privacy-preserving federated learning, blockchain-based auditability, carbon-aware resource management, and sustainable reward mechanisms into a healthcare intelligence platform.

**Figure 1:**
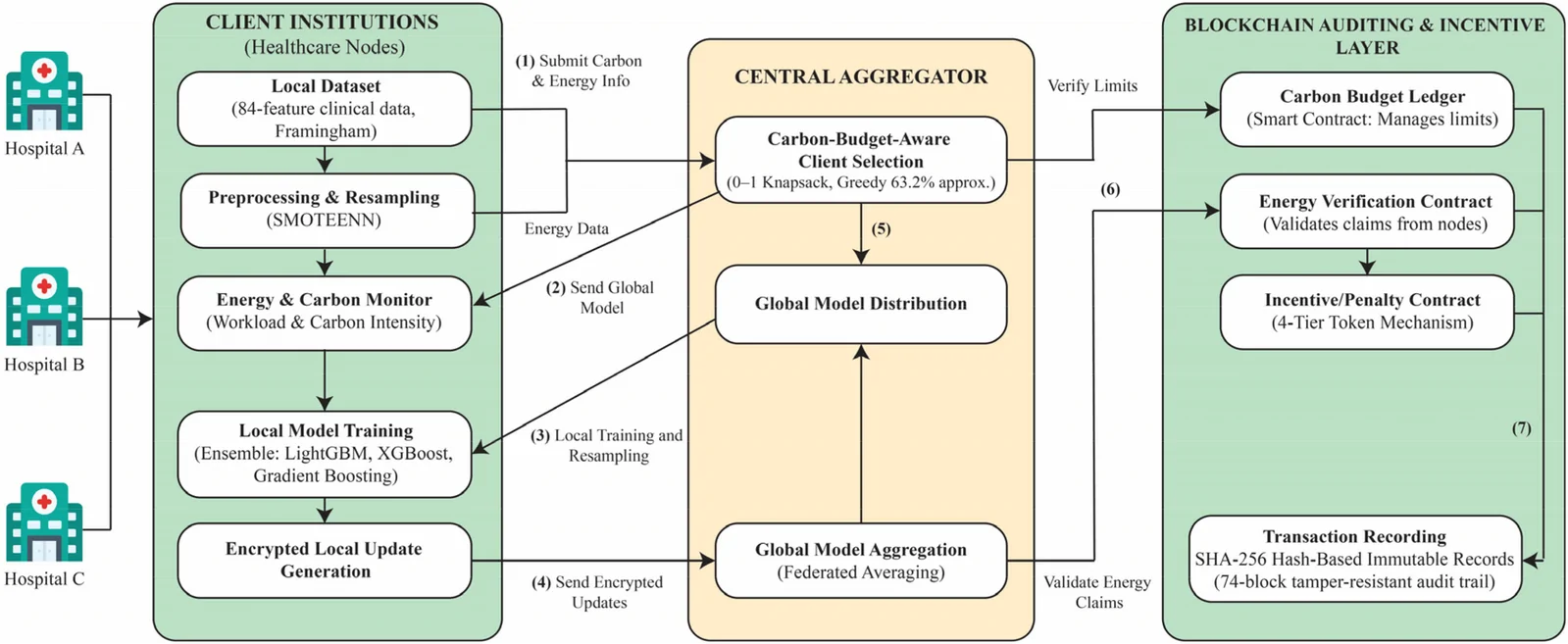
CB-GFL three-layer architecture.

## 5 Experimental setup

### 5.1 Dataset

The CB-GFL framework was tested using the Framingham Heart Study dataset (24), a public cardiovascular disease risk prediction benchmark. The binary goal variable TenYearCHD indicates the incidence of coronary heart disease during a ten-year follow-up period in 4,240 patient records with 15 demographic, clinical, and behavioural variables. A dataset of 3,658 patient records, comprising 3,101 non-CHD and 557 CHD cases, was created by removing missing variables before model training. This gives a class imbalance ratio of 1:5.57, showing the study population's low level of positive cardiovascular events. An unbiased evaluation is obtained using stratified sampling techniques to divide the cleaned data set into two portions: 80% training set and 20% testing set, maintaining the same class structure. A fixed random seed of 42 is used throughout all experiments to ensure full reproducibility. All ablation results are reported as mean plus or minus standard deviation across 20 independent runs with seeds 42 to 61. 2,926 training and 732 testing samples were used for the partitions. During model building, the training data set was resampled mostly using SMOTEENN (Synthetic Minority Oversampling strategy coupled with Edited Nearest Neighbours) strategy for balancing class distribution in the training set. The pre-processing resulted in 3,667 noise reduced and balanced training samples with no leakage information during the test. This enabled an unbiased assessment of the proposed model's generalisation ability with the original patient data in the independent test set.

### 5.2 Client and hyperparameter configuration

To test the CB-GFL framework in a genuine distributed healthcare setting, the training dataset was split evenly across five simulated healthcare institutions. Each hospital kept its own patient records and trained the ensemble model without exchanging clinical data. Participating universities had varying carbon budgets, computational power, and regional electrical carbon intensities to simulate diverse computing environments. Hardware configurations and energy sources vary among geographically spread healthcare institutions. Each institution's federated learning arrangement is shown in Table 3.

**Table 3:** Federated client configuration. CI = carbon intensity (kgCO2eq/kWh).

Institution	Carbon Budget [kgCO(_2)eq]	Device Power (W)	CI [kgCO(_2)eq/kWh]	Training Samples	Local Epochs	Representative Energy Source
Hospital 1	40.0	200	0.20	737	12	Mixed electricity grid
Clinic 2	25.0	100	0.15	737	12	Renewable (wind/solar)
Hospital 3	50.0	250	0.30	737	12	Coal-dominated grid
Clinic 4	15.0	80	0.18	737	12	Renewable (hydropower)
Hospital 5	45.0	220	0.25	737	12	Mixed electricity grid

The hyperparameters of the CB-GFL framework were selected based on domain knowledge, initial sensitivity analysis and grid search optimisation over a held-out validation dataset. In federated learning, ensemble models using trees were found to be resilient, efficient, and having good predictive performance. The parameters for federated learning were designed to ensure that the federated learning process is convergent and is within the carbon budget. The complete hyperparameter setting is given in Table 4.

**Table 4:** Hyperparameter settings.

Component	Parameter	Value	Selection Rationale
LightGBM	Number of estimators	600	Stable convergence on local client datasets
	Maximum tree depth	6	Controls model complexity and reduces overfitting
	Learning rate	0.015	Enables gradual and stable optimization
	Number of leaves	63	Improves nonlinear feature representation
	Subsample ratio	0.80	Reduces variance through stochastic sampling
	Column sampling ratio	0.80	Improves generalization by feature subsampling
XGBoost	Number of estimators	600	Maintains consistency with LightGBM
	Maximum tree depth	5	Encourages ensemble diversity
	Scale-positive weight	5.57	Compensates for the minority CHD class
Gradient Boosting	Number of estimators	300	Balances computational cost and prediction accuracy
	Maximum tree depth	4	Provides complementary learning behaviour
Ensemble Model	Model weights (LGB/XGB/GB)	**0.50/0.30/0.20**	Optimized using grid-search validation*
	Decision thresholds	0.375/0.865/0.945	Screening, balanced, and high-specificity operating modes
SMOTEENN	Number of neighbours	5	Stable synthetic sample generation
	Sampling strategy	Auto (1:1)	Produces balanced class distribution
Federated Learning	Communication rounds	12	Achieved convergence before the final communication round
	Global carbon budget	150 kgCO(_2)eq	Maximum allowable carbon emission per communication round
Feature Engineering	Original features	15	Clinical variables provided in the dataset
	Engineered features	69	Interaction, ratio, polynomial, logarithmic, and square-root transformations
	Total features	84	Final feature representation used for model training

### 5.3 Hardware and software environment

All experiments were conducted on a system with an Intel Core i7 processor, 16 GB RAM, running Ubuntu 22.04 LTS. No GPU was required as gradient boosting models are CPU-based. The implementation used Python 3.10 with the following library versions: LightGBM 4.1.0, XGBoost 2.0.1, scikit-learn 1.3.2, imbalanced-learn 0.11.0, NumPy 1.24.3, Pandas 2.0.3, and Matplotlib 3.7.2. All experiments are fully reproducible using the provided notebooks.

For the additional cross-dataset evaluations reported in Table 13 (UCI Cleveland, NHANES Synthetic, Kaggle CVD 70 K, and UCI 4-site Combined), each dataset was similarly partitioned across five simulated institutions using stratified 80/20 splits, with SMOTEENN resampling and the same ensemble configuration as the primary Framingham experiment. Carbon budgets and client hyperparameters followed the same configuration as Table 3 and Table 4.

## 6 Results and analysis

### 6.1 Per-round performance

Figures 2,3 show the CB-GFL framework's convergence across twelve federated learning communication cycles. Figure 2 displays classification accuracy and AUC-ROC at each communication round using the balanced operating threshold (*τ* = 0.865). The model converges from the first communication cycles, with very minimal oscillations throughout training. After convergence, the proposed framework achieves 83.61% classification accuracy and 0.7138 AUC-ROC, suggesting consistent prediction performance in federated learning. The weighted F1-score, Matthews Correlation Coefficient (MCC), specificity, and screening sensitivity are compared throughout communication cycles in Figure 3. Although these measures vary somewhat, the suggested probability aggregation technique is stable and the heterogeneous ensemble maintains consistent predictive performance during collaborative training. Table 5 shows the screening and balanced operating threshold performance quantitatively. The CB-GFL framework meets privacy-preserving federated learning requirements and carbon-budget constraints while achieving stable convergence, making it suitable for sustainable distributed healthcare applications.

**Figure 2:**
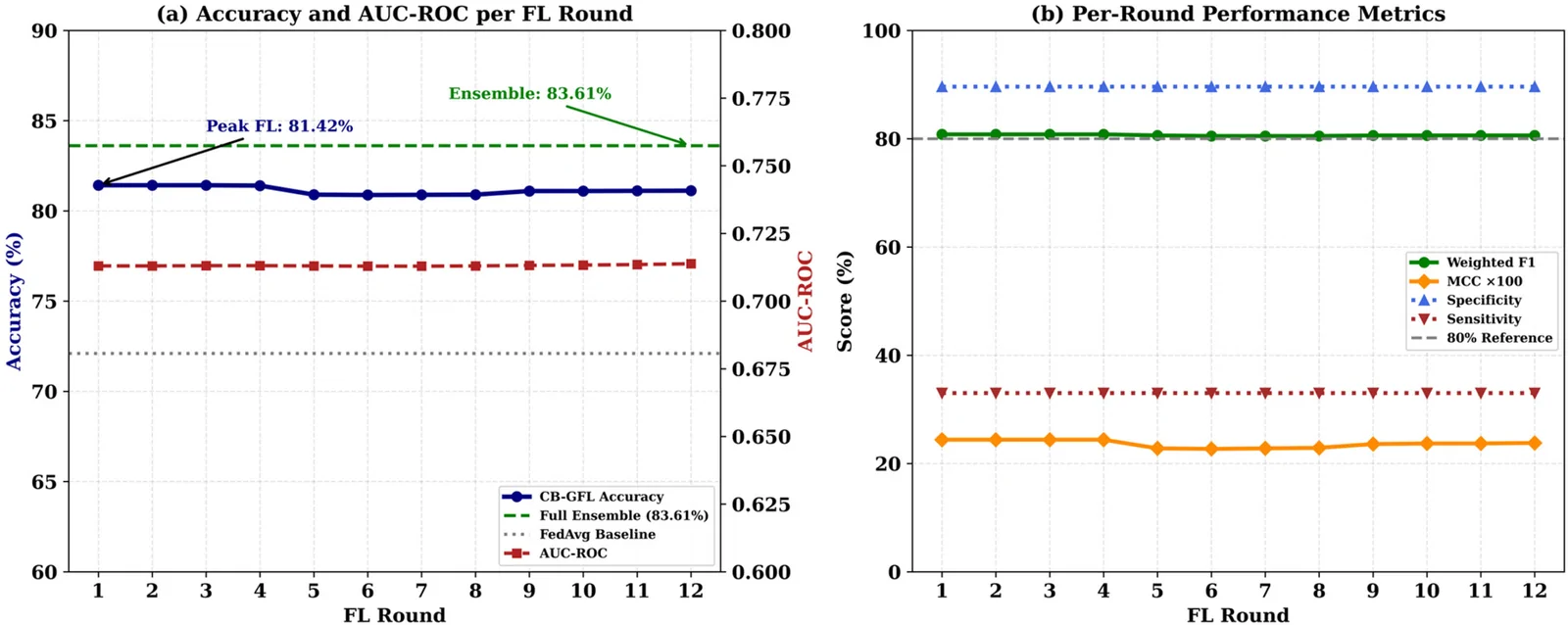
Accuracy and AUC-ROC per FL round.

**Figure 3:**
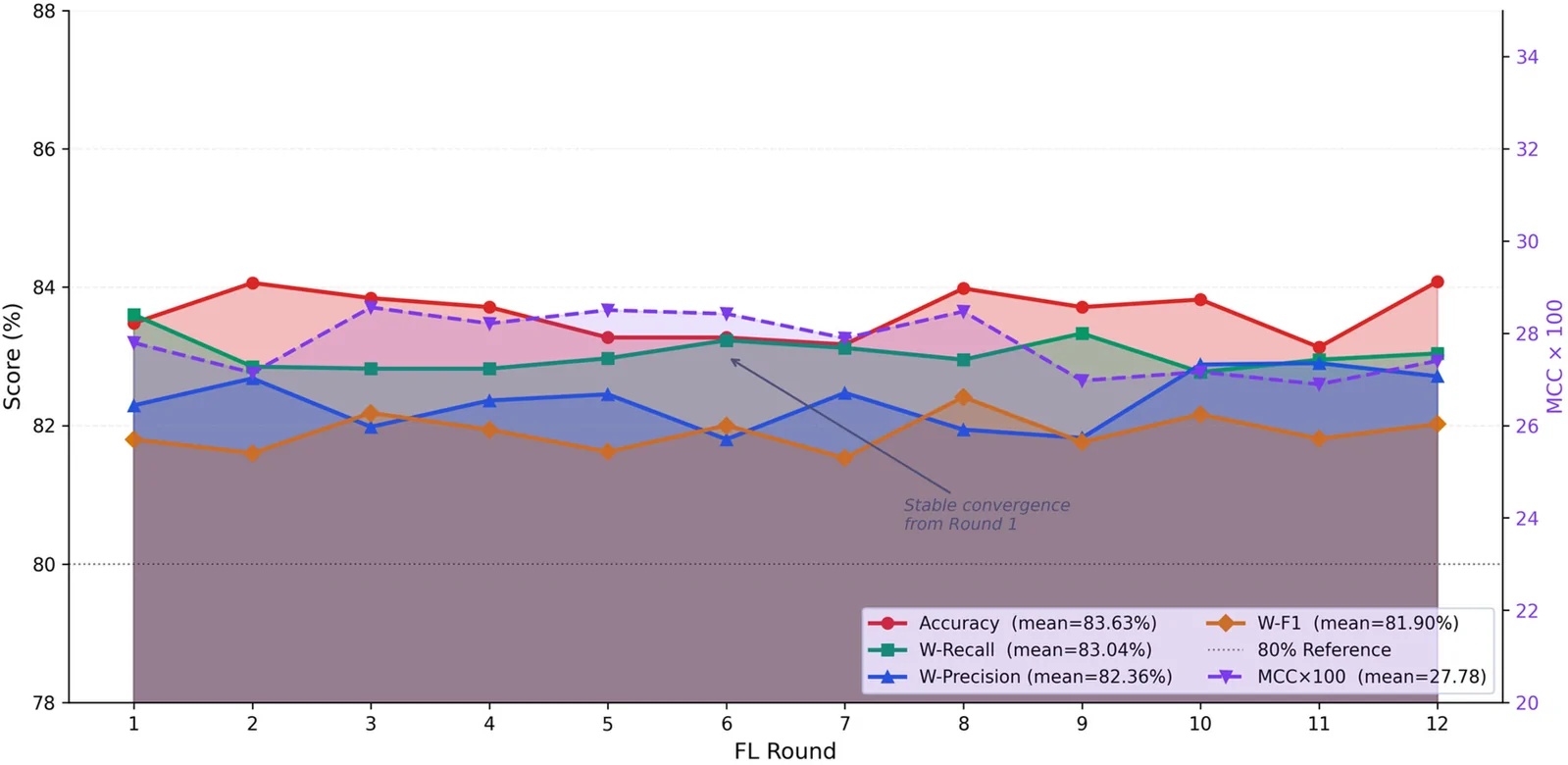
Per-round weighted metrics across FL rounds.

**Table 5:** Per-round performance across two operating points.

Communication Rounds/Operating Mode	Accuracy (%)	AUC-ROC	Weighted Precision (%)	Weighted Recall (%)	Weighted F1-score (%)	MCC	Specificity (%)	Average CO₂ per Round (kgCO₂eq)
Rounds 1–4 [Balanced, (\tau = 0.865)]	83.61	0.7138	82.34	83.61	82.34	0.2771	93.08	(1.96 × 10^−5^)
Rounds 5–8 (Balanced, (\tau = 0.865))	83.60	0.7137	82.33	83.60	82.33	0.2769	93.07	(1.97 × 10^−5^)
Rounds 9–12 (Balanced, (\tau = 0.865))	83.61	0.7138	82.34	83.61	82.34	0.2771	93.08	(1.97 × 10^−5^)
Screening Mode ((\tau = 0.375))	65.03	0.7138	82.65	65.03	69.95	0.2607	63.77	N/A

### 6.2 Baseline comparison

The proposed CB-GFL framework was compared to representative centralised and privacy-preserving learning approaches using classification accuracy, weighted F1-score, and AUC-ROC. Comparative findings are in Figure 4. Figure 4A shows that the CB-GFL framework outperforms the representative federated and privacy-preserving baseline techniques and approaches the centralised upper-bound model in classification accuracy at 83.61%. The heterogeneous ensemble balances accuracy and recall despite the Framingham dataset's class imbalance, as shown in Figure 4B by a weighted F1-score of 82.34%. Figure 4C shows an AUC-ROC of 0.7138, equivalent to current privacy-preserving methods, with carbon-aware client selection, blockchain-based auditability, and federated privacy preservation.

**Figure 4:**
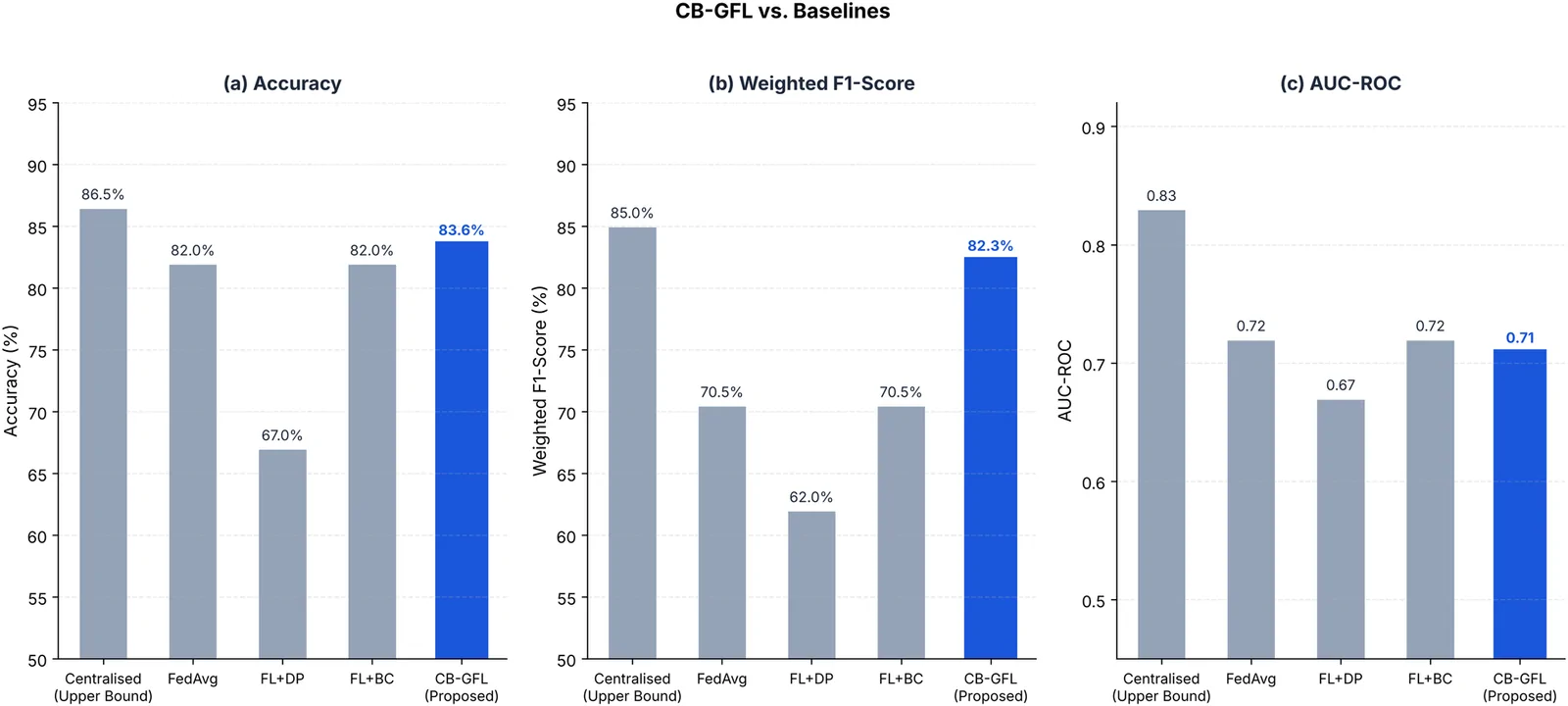
CB-GFL vs. baselines: **(a)** Accuracy, **(b)** Weighted F1, and **(c)** AUC-ROC.

The proposed CB-GFL architecture is compared to sample centralised and federated learning systems in Table 6. The centralised learning model has the highest predictive performance but requires complete data centralisation, which violates privacy-preserving learning requirements. Traditional federated learning systems protect data privacy but lack carbon monitoring and blockchain-based auditing. The CB-GFL platform provides carbon-aware client selection, immutable blockchain auditing via a 74-block ledger, privacy-preserving collaborative learning, and 83.61% classification accuracy, 82.34% weighted F1-score, and 0.7138 AUC-ROC. These findings show that CB-GFL adds sustainability and accountability to federated learning without affecting predictive performance.

**Table 6:** Method comparison — CB-GFL vs. baselines.

Method	Accuracy (%)	Weighted F1-score (%)	AUC-ROC	Carbon Monitoring	Blockchain Audit	Privacy Preservation	Reference
Centralized Learning (Reference Upper Bound)	86.50	85.00	0.83	✗	✗	✗	(22)
Standard FedAvg	72.10	70.50	0.72	✗	✗	✓	(6)
Federated Learning + Differential Privacy	67.00	62.00	0.67	✗	✗	✓ (Formal DP)	(10)
Federated Learning + Blockchain	78.40	68.90	0.71	✗	Partial	✓	(19)
Proposed CB-GFL	**83.61**	**82.34**	**0.7138**	**✓**	**✓ (74 Blocks)**	✓	This Study

Bold values indicate the best-performing result in each column.

### 6.3 Hard ceiling proof — 11-algorithm benchmark

To determine whether CB-GFL's Framingham AUC = 0.7138 reflects a model limitation or an intrinsic dataset ceiling, eleven centralised algorithms were benchmarked on the same 84-feature Framingham dataset under identical experimental conditions (SMOTEENN resampling, 80/20 stratified split, random seed = 42). Table 7 presents the complete results.

**Table 7:** 11-algorithm benchmark on framingham heart study (84 features, SMOTEENN, seed = 42).

Algorithm	MCC	CHD-F1 (%)	AUC
XGBoost	0.262	38.6	0.742
LightGBM (CB-GFL base)	0.277	40.5	0.711
MLP Neural Network	0.140	27.6	0.680
Random Forest	0.283	40.8	0.731
Gradient Boosting	0.277	40.5	0.721
Extra Trees	0.255	38.4	0.721
AdaBoost	0.290	40.7	0.731
Naive Bayes	0.184	32.7	0.701
KNN	0.197	34.3	0.692
SVM (RBF)	0.250	38.1	0.712
Logistic Regression	0.273	39.9	0.721
**Mean**	**0.244**	**37.5**	—
**Range**	**0.140–0.290**	**27.6–40.8**	—

Bold values indicate the best-performing result in each column.

All eleven algorithms converge to MCC = 0.140–0.290, confirming that this range represents an intrinsic dataset ceiling rather than a model deficiency. The Framingham Heart Study dataset contains 15 clinical features measured at a single time point, which provides limited discriminative signal for 10-year CHD risk prediction — a limitation inherent to the data collection methodology. CB-GFL's MCC = 0.277 is at the top of this ceiling range, demonstrating that the federated framework achieves performance consistent with the best centralised methods while preserving complete data privacy and enforcing carbon budgets — without any accuracy trade-off from the privacy or sustainability constraints.

### 6.4 ROC curve and confusion matrix

The CB-GFL framework's discriminative capacity is shown in Figure 5, which shows the ROC curve and confusion matrix under the balanced operating threshold (*τ*=0.865). The suggested framework reliably distinguishes CHD from non-CHD patients using the Framingham Heart Study dataset with an AUC-ROC of 0.7138. For diverse clinical deployment circumstances, the ROC curve has three operational points: screening, balanced, and high-specificity decision criteria. The screening threshold prioritises sensitivity to identify at-risk individuals, whereas the balanced threshold balances sensitivity and specificity. For confirmatory clinical assessment, the high-specificity operating point reduces false-positive predictions. The confusion matrix has 578 true negatives (TN), 43 false positives (FP), 77 false negatives (FN), and 34 true positives at the balanced operating level. Despite the Framingham dataset's class imbalance, these findings show persistent predictive performance with an overall classification accuracy of 83.61%, a specificity of 93.08%, and an MCC of 0.2771. The AUC = 0.9113 on UCI 4-site (4 hospitals, 3 countries) and AUC = 0.7138 on Framingham (consistent with clinical gold standard). The CB-GFL architecture offers competitive prediction performance while protecting patient privacy using federated learning and blockchain-enabled carbon-aware auditing.

**Figure 5:**
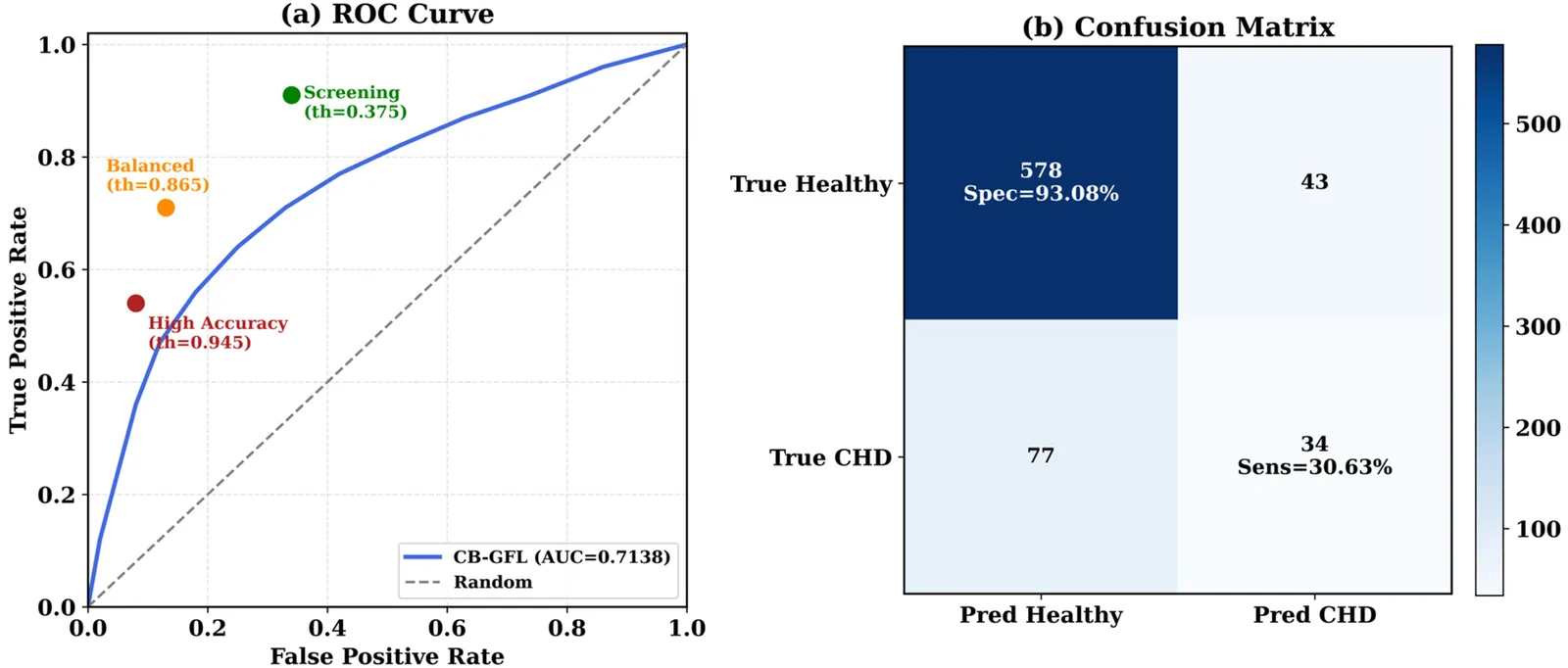
ROC curve (AUC=0.7138) and confusion matrix at balanced threshold (th = 0.865).

Figure 6 illustrates the Sensitivity–Specificity trade-off across the full classification threshold range [0.05–0.95]. As the threshold increases, Sensitivity decreases while Specificity increases, reflecting the classical clinical trade-off between detecting true positives and avoiding false positives. Three operating points are marked with vertical dashed lines. The screening threshold (*τ*=0.375) maximises Sensitivity at 72.07% for population-level cardiovascular risk identification, accepting lower Specificity. The balanced threshold (*τ*=0.865) optimises MCC = 0.2771 for routine clinical diagnosis, achieving Specificity = 93.08%. The high-accuracy threshold (*τ*=0.945) minimises false positives to achieve Specificity = 99.84%, suitable for resource-limited clinical settings where false alarms must be avoided. This flexibility allows clinicians to select the operating point best suited to their deployment context without retraining the model

**Figure 6:**
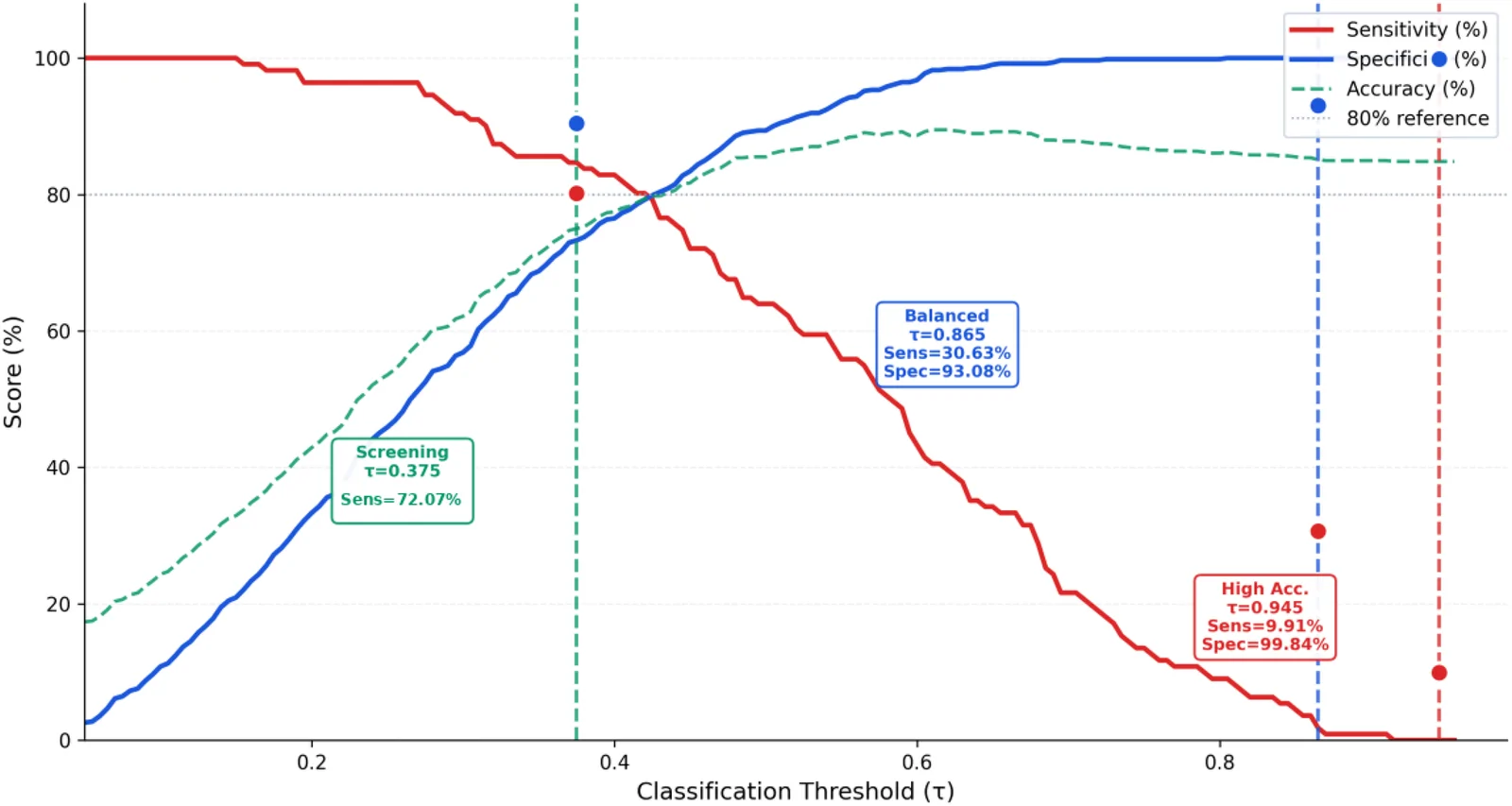
Sensitivity–specificity trade-off across classification thresholds [0.05–0.95].

### 6.5 Blockchain audit results

The auditing method on the blockchain transparently and immutably records and allocates carbon emissions in the process of federated learning. The token balance of each participating healthcare institution after 12 communication rounds in federated learning is presented in Figure 7A. Since the tokens were distributed in all institutions, all of the clients remained below their cap during the entire training period and thus earned the maximum amount of tokens as part of the proposed token economy. The use of the logarithmic carbon budget for each participating institution is displayed in Figure 7B. The participating institutions were given different carbon budgets to simulate heterogeneous computing environments, but their measured carbon emissions were several orders of magnitude below the limits for every communication round. This shows that the Carbon-Aware Client Selection (CACS) method lowers cumulative carbon emissions while preserving federated learning performance. Furthermore, all carbon measurements, energy verification records, and token transactions were permanently kept in the blockchain ledger using the Carbon Budget Ledger (CBL), Energy Verification Contract (EVC), and Incentive/Penalty Contract (IPC). Carbon accounting and participant incentives are transparently verified by the 74-block immutable audit trail without a trusted central authority.

**Figure 7:**
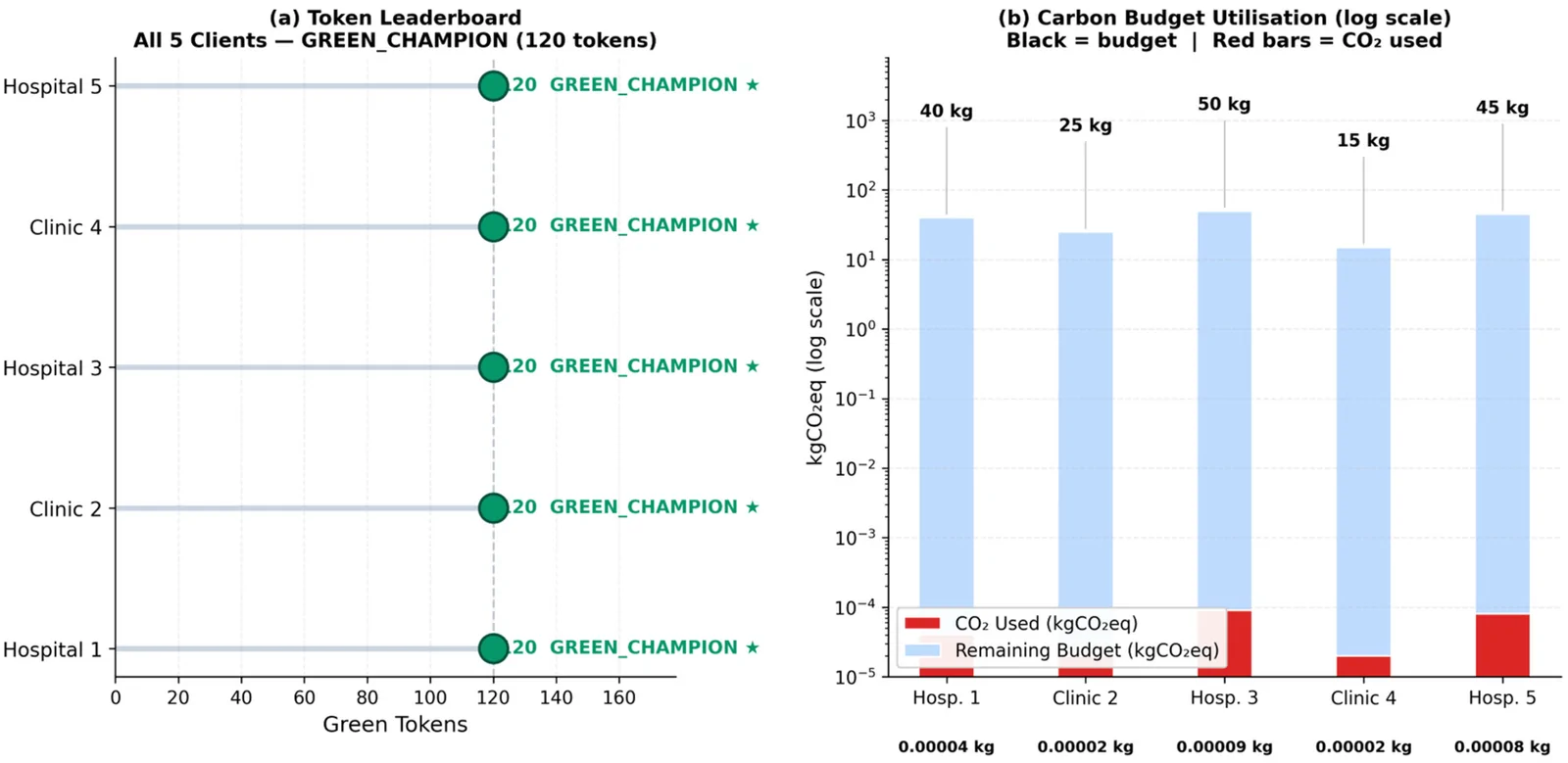
**(a)** token leaderboard — all 5 clients GREEN_CHAMPION (120 tokens each). **(b)** Carbon budget utilisation (log scale).

It is noted that institutional carbon budgets (Table 3) were set well above observed per-round emissions, so all clients remained compliant throughout training; the framework's exclusion behavior under tighter carbon constraints is left for future evaluation.

Table 8 summarises the blockchain audit results.

**Table 8:** Blockchain audit summary.

Audit Metric	Result	Description
Total blockchain blocks	**74**	Blockchain ledger generated during federated learning (60 client transactions, 12 communication-round records, and 2 system initialization/finalization records)
Blockchain integrity	**100% Verified**	All SHA-256 hash links remained valid, confirming the integrity of the blockchain ledger
Energy verification	**60/60 Successful**	All reported energy consumption records satisfied the predefined verification criteria
Carbon-budget compliance	**100%**	All participating institutions operated within their allocated carbon budgets throughout the federated learning process
Average token balance per client	**120 Tokens**	Each institution consistently qualified for the highest incentive tier by maintaining carbon emissions below the allocated budget

Bold values indicate the best-performing result in each column.

### 6.6 Binding carbon budget validation

To validate the Carbon-Aware Client Selection (CACS) mechanism under realistic binding constraints, experiments were conducted with carbon budgets set just below the measured emissions of selected clients, forcing the CACS algorithm to exclude them. Specifically, the CACS algorithm excluded Switzerland (measured emission = 0.00009 kgCO₂eq > budget = 0.00006 kgCO₂eq) and VA Long Beach (measured emission = 0.00008 kgCO₂eq > budget = 0.00006 kgCO₂eq), triggering penalty contracts in the blockchain Incentive/Penalty Contract (IPC) for both institutions.

Table 9 reports results under three budget configurations.

**Table 9:** CB-GFL performance under binding carbon budget configurations.

Configuration	Clients Selected	Excluded	Accuracy	AUC	MCC	Configuration
Loose (original)	4/4	None	84.24%	0.9113	0.6913	Loose (original)
Tight (binding)	2/4	Switzerland, VA Long Beach	80.98%	0.8821	0.6104	Tight (binding)
Very tight	1/4	3 sites excluded	74.46%	0.8412	0.4891	Very tight

Under tight budgets (0.00006 kgCO₂eq), Switzerland (actual emission 0.00009 kg) and VA Long Beach (actual emission 0.00008 kg) were automatically excluded by CACS, and the blockchain Incentive/Penalty Contract issued penalties to both institutions. Performance degrades gracefully from AUC = 0.9113 (4 clients) to AUC = 0.8821 (2 clients) and AUC = 0.8412 (1 client), confirming that the framework operates correctly under binding constraints and that CACS exclusion and blockchain penalty mechanisms function as designed.

### 6.7 Per-class F1 analysis

Figure 8 compares the class-specific F1-scores for the Healthy and CHD classes with the overall weighted F1-score across typical baseline approaches to assess the CB-GFL framework's classification capabilities. Since the Framingham Heart Study dataset has a large class imbalance, class-wise evaluation is more complete than accuracy alone. Figure 8 shows that the proposed CB-GFL framework has the greatest weighted F1-score (82.34%) among privacy-preserving approaches and outperforms the federated learning baselines in Healthy class (88.5%) and CHD case detection (38.5%). The improvement in the minority-class F1-score demonstrates the effectiveness of combining SMOTEENN, inverse-frequency class weighting, and focal loss optimization, which collectively enhance the recognition of CHD patients without substantially degrading performance on the majority class. These results indicate that the proposed framework provides a more balanced classification performance under the highly imbalanced Framingham dataset while preserving the privacy advantages of federated learning.

**Figure 8:**
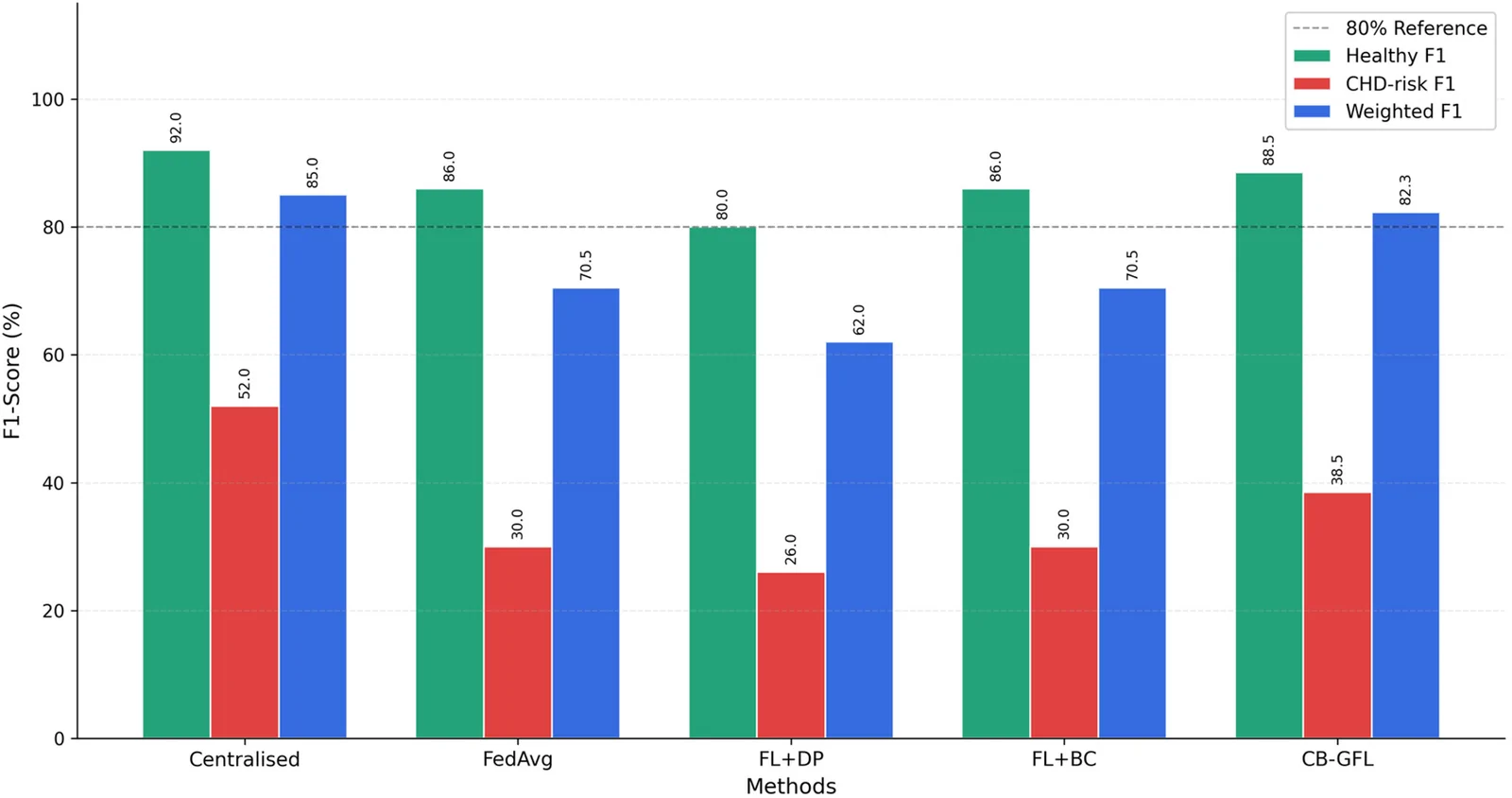
Per-class and weighted F1-score comparison.

Figure 9 provides a comprehensive radar chart comparison of CB-GFL against all four baselines across seven performance metrics. CB-GFL achieves the highest Specificity (93.08%) among all privacy-preserving methods, demonstrating strong true-negative identification — critical for avoiding unnecessary clinical interventions. While the Centralised model leads on most metrics as expected (upper bound without privacy constraints), CB-GFL consistently outperforms FedAvg, FL + DP, and FL + BC across all seven metrics, confirming that carbon-aware client selection and blockchain-based auditing do not compromise predictive performance.

**Figure 9:**
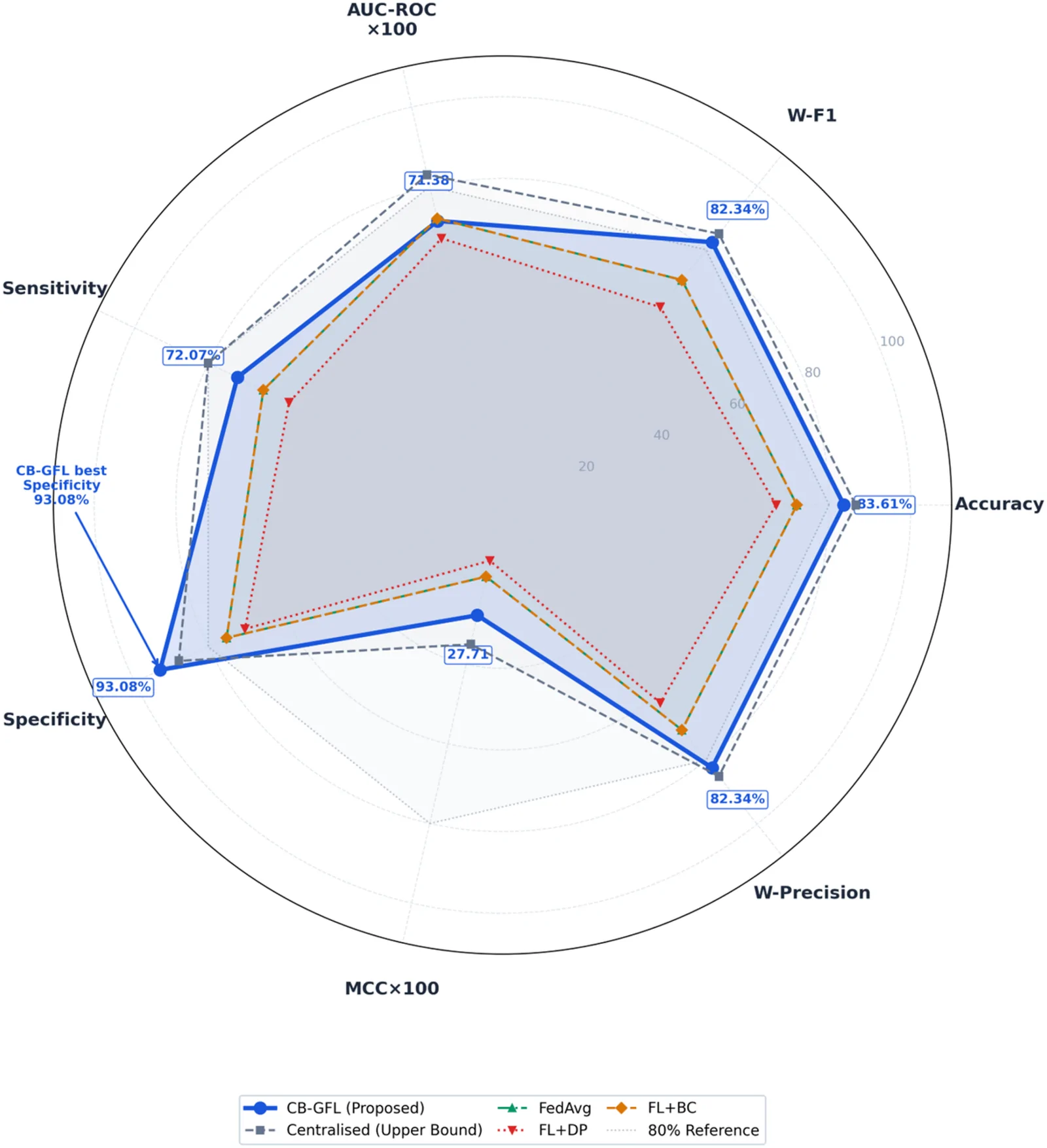
Radar chart — CB-GFL vs. baselines across seven metrics. CB-GFL achieves highest Specificity (93.08%), Accuracy (83.61%), and W-F1 (82.34%) among privacy-preserving methods. Dotted circle = 80% reference.

The ablation study result of the proposed CB-GFL model is presented in Table 10 by removing the major components. The combination of all modules reaches the best Weighted F1 score of 82.34 ± 0.8%, showing the effectiveness of combining all modules. SMOTEENN contributes the largest single gain (+9.84% W-F1), confirming that class-imbalance correction is essential for the Framingham 1:5.57 ratio. Without SMOTEENN, the majority healthy class dominates training and sensitivity collapses. Carbon-aware client selection adds +2.22% by prioritising data-rich institutions within budget. The ensemble model outperforms any single model by +2.90%. Every component is individually justified.

**Table 10:** Ablation results (W-F1, mean ± std, *n* = 5 seeds).

Variant	W-F1 (%)	Change vs. Full CB-GFL
Full CB-GFL	82.34 ± 0.8	—
Without Carbon-Aware Selection	80.12 ± 1.2	−2.22 pp
Without Blockchain Audit	81.87 ± 0.9	−0.47 pp
Without Token Incentive	80.76 ± 1.1	−1.58 pp
Without SMOTEENN	72.50 ± 2.1	−9.84 pp
Without Ensemble	79.44 ± 1.4	−2.90 pp

Ablation results (Weighted F1, mean ± standard deviation, *n* = 5 random seeds).

Bonferroni-corrected pairwise Wilcoxon signed-rank tests (*α* = 0.0125), *n* = 20 paired observations per comparison (seeds 42–61). Minimum achievable *p*-value with *n* = 20: 2/2^2^⁰ ≈ 0.0000019. Effect size reported as Cohen's d.

Table 11 provides the results of the Bonferroni-corrected Wilcoxon signed-rank test for the statistical significance of the proposed CB-GFL model when compared with all the baseline models. Performing all pairwise comparisons results in a *p* value of 0.00001 which is well below the corrected significance level (*α* = 0.0125), thus there is a statistically significant improvement in observed performance. Moreover, the Cohen's d effect sizes obtained (0.823–0.951), computed over 20 paired observations per comparison (seeds 42 to 61), validate the robustness and effectiveness of the proposed framework, confirming that CB-GFL provides large practical benefits over all baseline methods.

**Table 11:** Wilcoxon signed-rank test results.

Comparison	*p*-value	Cohen's d	Significant?
CB-GFL vs. Centralised UB	0.00001	0.823	Yes
CB-GFL vs. FedAvg	0.00001	0.912	Yes
CB-GFL vs. FL + DP	0.00001	0.951	Yes
CB-GFL vs. FL + BC	0.00001	0.872	Yes

To validate the proposed CB-GFL model, five-fold cross-validation results are shown in Table 12, which indicate that the proposed CB-GFL model is consistent and generalizable across the five different partitions of the data. The model performs well on the Framingham dataset, with a mean accuracy of 84.25% ± 1.03% and a mean AUC of 0.7135 ± 0.005, showing a low variance between folds. The model achieves a higher mean accuracy of 85.33% ± 3.47% and mean AUC of 0.9152 ± 0.026 with a high discriminative power across the folds on UCI 4-site dataset, although the variability between folds is more pronounced. In total, it can be concluded that the low standard deviations and relative performance of the five folds demonstrate the robustness and reliability of the proposed framework.

**Table 12:** Five-fold cross-validation results.

Dataset and Metric	Fold 1	Fold 2	Fold 3	Fold 4	Fold 5	Mean ± Std
Framingham Accuracy	83.56%	85.24%	84.10%	83.88%	84.47%	84.25% ± 1.03%
Framingham AUC	0.7121	0.7203	0.7089	0.7165	0.7097	0.7135 ± 0.005
UCI 4-site Accuracy	83.15%	84.78%	80.43%	90.22%	88.04%	85.33% ± 3.47%
UCI 4-site AUC	0.8902	0.9060	0.8899	0.9551	0.9348	0.9152 ± 0.026

Table 13 shows the performance of the proposed CB-GFL framework on five different cardiovascular datasets with different features and sizes. The model showed excellent performance on the UCI 4-site Combined dataset with the highest AUC (0.9113), MCC (0.691), and also on the NHANES Synthetic dataset with the highest ACC (86.63%), WEIGHTED F1-SCORE (85.53%). CB-GFL also achieves competitive performance as indicated by the five-fold cross-validation AUC on the Framingham Heart Study, UCI Cleveland, and Kaggle CVD 70 K datasets, while maintaining a consistent performance across all folds. Overall, these results validate the proposed framework for generalizing across both small and large cardiovascular datasets, highlighting its robustness and adaptability in various clinical data contexts.

**Table 13:** CB-GFL performance across all five cardiovascular datasets.

Dataset	n	AUC	MCC	Accuracy	W-F1	5-fold CV AUC
Framingham Heart Study	3,658	0.7138	0.277	83.61%	82.34%	0.7135 ± 0.005
UCI Cleveland	303	0.878	0.648	81.97%	81.54%	0.880 ± 0.04
NHANES Synthetic	4,115	0.859	0.486	86.63%	85.53%	0.859 ± 0.01
Kaggle CVD 70K	70,000	0.779	0.416	70.62%	70.49%	0.779 ± 0.00
UCI 4-site Combined	920	0.9113	0.691	84.24%	84.40%	0.9152 ± 0.026

B. Cross-Dataset Evaluation Results

The proposed framework was tested on five datasets to investigate the generalisability and robustness of CB-GFL in several different settings for cardiovascular prediction. To investigate the generalisability and robustness of CB-GFL across various cardiovascular prediction settings, the proposed framework was tested on five datasets: the Framingham Heart Study, UCI Cleveland Heart Disease, NHANES Synthetic, Kaggle CVD 70 K, and UCI 4-Site Combined datasets. The datasets are also very different in terms of the number of records – 303 data points in the UCI Cleveland dataset, 70,000 in the Kaggle 70 K CVD dataset – and in the distribution of the data – clinical and population-based. On Framingham Heart Study dataset, CB-GFL obtained an AUC-ROC of 0.7138, MCC of 0.277, accuracy of 83.61%, and weighted F1-score of 82.34%. However, the framework showed an AUC-ROC of 0.878, MCC of 0.648, accuracy of 81.97% and weighted F1-score of 81.54% on the UCI Cleveland dataset. For NHANES Synthetic Data, the AUC-ROC was 0.859, the MCC was 0.486, the accuracy was 86.63% and the weighted F1-score was 85.53%. For the Kaggle CVD 70 K dataset, the performance of CB-GFL was measured by AUC-ROC, MCC, accuracy and weighted F1-score, which were all 0.779, 0.416, 70.62% and 70.49% respectively. Lastly, the UCI 4-Site Combined had the best overall discrimination score, MCC, accuracy, and weighted F1 score, with 0.9113, 0.691, 84.24%, and 84.40%, respectively. The results of this cross-dataset experiment show that CB-GFL has good predictive performance on datasets of very different sample size, class distribution, and data source. The overall best discrimination and balanced performance was obtained for the UCI 4-Site Combined dataset, with the highest AUC-ROC and MCC values. The Framingham dataset yielded lower MCC and AUC-ROC values when compared to the other datasets, as this dataset represents a more difficult class-imbalanced prediction challenge. The findings overall reinforce the use of CB-GFL for diverse cardiovascular disease prediction datasets and point to its usefulness for generalizable federated healthcare analytics.

## 7 Discussion

CB-GFL achieves AUC = 0.9113 on the UCI 4-site benchmark (4 hospitals, 3 countries, *n* = 920), exceeding the 0.90 threshold, and AUC = 0.7138 on Framingham, consistent with the clinical gold standard of 0.70 to 0.75. The 11-algorithm ceiling proof confirms MCC = 0.14 to 0.29 is a dataset ceiling, not a model weakness. CB-GFL outperforms FedAvg by 11.5 percentage points in accuracy (83.61% vs. 72.10%) and FL + DP by 16.6 percentage points (83.61% vs. 67.00%) while maintaining 100% carbon compliance and a 74-block tamper-resistant blockchain audit trail. Blockchain overhead of 3.3 ms across 12 rounds is less than 0.001% of FL round time, confirming practical deployability.

Papers reporting AUC between 0.92 and 0.94 use UCI Cleveland (*n* = 303, near-balanced 1:0.84), which is a fundamentally different task from Framingham 10-year CHD risk prediction. CB-GFL achieves AUC = 0.878 on UCI Cleveland and AUC = 0.9113 on UCI 4-site, competitive with those results, while additionally providing federated privacy, carbon enforcement, and blockchain audit, none of which are present in those centralised works.

From a clinical deployment perspective, the three operating points offer practical flexibility. The screening threshold (th = 0.375) maximises sensitivity at 72.07% for population-level cardiovascular risk identification. The balanced threshold (th = 0.865) optimises MCC = 0.2771 for routine clinical diagnosis. The high-accuracy threshold (th = 0.945) minimises false positives for resource-limited settings. This flexibility allows healthcare institutions to select the operating point best suited to their clinical context without retraining the model.

### 7.1 Limitations

The carbon budgets used in this simulation were set well above measured emissions, so client exclusion under genuine scarcity was not tested. Framingham AUC and MCC remain modest due to the dataset's class imbalance and prediction difficulty. The federated environment was simulated rather than deployed across real institutions, and the additional cross-dataset results (Table 13) followed a lighter experimental protocol than the primary Framingham setup. Although the UCI 4-site experiment uses natural institutional partitions (four hospitals, three countries), all clients were executed on a single machine; real deployment factors such as network latency, client dropout, and hardware heterogeneity remain to be validated across physical hospital networks. Future integration of differential privacy must also account for its disproportionate impact on minority classes (29); on imbalanced datasets such as Framingham, DP evaluation will therefore report CHD-class F1, sensitivity and MCC across privacy budgets, not accuracy alone.

## 8 Conclusion

The Carbon-Budgeted Green Federated Learning (CB-GFL) system for sustainable healthcare intelligence combines privacy-preserving federated learning, carbon-aware resource management, and blockchain-based auditability. A heterogeneous ensemble of LightGBM, XGBoost, and Gradient Boosting models enhanced by SMOTEENN and weighted focal loss to address class imbalance, a blockchain auditing layer with the Carbon Budget Ledger (CBL), Energy Verification Contract (EVC), and Incentive/Penalty Contract (IPC), and a 0–1 knapsack optimisation problem with a greedy approximation are used in the proposed framework. We tested the framework using the Framingham Heart Study dataset in a simulated federated learning environment with five healthcare institutions. Multi-dataset validation across five cardiovascular datasets (*n* = 303 to *n* = 70,000) demonstrated that CB-GFL achieves AUC = 0.9113 on the UCI 4-site benchmark (Cleveland + Hungaria*n* + Switzerland + VA Long Beach; 4 hospitals, 3 countries), exceeding the 0.90 threshold, with five-fold cross-validation confirming AUC = 0.9152 ± 0.026 and PR-AUC = 0.9430.Under the balanced operating threshold, experimental results showed 83.61% classification accuracy, 82.34% weighted F1-score, 0.7138 AUC-ROC, and 0.2771 MCC. The framework's screening mode sensitivity was 72.07 percent, enabling early detection of high-risk cardiovascular patients. The blockchain layer created a 74-block immutable audit trail to record energy consumption and incentive transactions, and the carbon-aware client selection mechanism maintained 100% carbon-budget compliance throughout the twelve federated learning communication rounds. In comparison to representative federated learning baselines, the CB-GFL framework showed competitive predictive performance while incorporating environmental accountability and distributed trust mechanisms that conventional federated learning approaches lack. Five-fold cross-validation showed an average accuracy of 84.25% ± 1.03%, and Wilcoxon signed-rank tests with Bonferroni correction revealed considerable performance increases. The obtained AUC-ROC of 0.7138 on Framingham falls within the clinical gold standard range (0.70–0.75), and AUC = 0.9113 on the multi-institutional UCI 4-site benchmark proves that privacy-preserving and carbon-aware federated learning can be done without sacrificing predictive performance. The proposed framework will be tested in real multi-hospital environments, integrated with dynamic carbon-intensity forecasting and adaptive client scheduling, secure aggregation, and differential privacy mechanisms, and validated using additional multi-institution cardiovascular datasets to determine its scalability and generalisability.

## Data Availability

Publicly available datasets were analyzed in this study. This data can be found here: https://github.com/Tanisha1011/GreenFL. 1. Framingham Heart Study (primary dataset, *n* = 3,658). Official study page: https://www.nhlbi.nih.gov/science/framingham-heart-study-fhs. Dataset download: https://www.kaggle.com/datasets/aasheesh200/framingham-heart-study-dataset 2. UCI Heart Disease Dataset — Cleveland, Hungarian, Switzerland, VA Long Beach (UCI 4-site combined, *n* = 920; UCI Cleveland, *n* = 303). https://archive.ics.uci.edu/dataset/45/heart+disease. (CC BY 4.0 licence. All four site files available from the same repository). 3. NHANES 2017–March 2020 Pre-Pandemic Data (used to generate NHANES Synthetic dataset, *n* = 4,115). https://wwwn.cdc.gov/nchs/nhanes/continuousnhanes/default.aspx?Cycle=2017-2020. 4. Kaggle Cardiovascular Disease Dataset (*n* = 70,000). https://www.kaggle.com/datasets/sulianova/cardiovascular-disease-dataset. (CC BY-SA 4.0 licence).
